# Anti-dsDNA Antibodies Promote Initiation, and Acquired Loss of Renal Dnase1 Promotes Progression of Lupus Nephritis in Autoimmune (NZBxNZW)F1 Mice

**DOI:** 10.1371/journal.pone.0008474

**Published:** 2009-12-29

**Authors:** Kristin Fenton, Silje Fismen, Annica Hedberg, Natalya Seredkina, Chris Fenton, Elin Synnøve Mortensen, Ole Petter Rekvig

**Affiliations:** 1 Department of Biochemistry, Institute of Medical Biology, Medical Faculty, University of Tromsø, Tromsø, Norway; 2 Department of Pathology, Institute of Medical Biology, Medical Faculty, University of Tromsø, Tromsø, Norway; 3 Department of Pathology, University Hospital of Northern Norway, Tromsø, Norway; 4 Department of Rheumatology, University Hospital of Northern Norway, Tromsø, Norway; 5 The Microarray Platform, Medical Faculty, University of Tromsø, Tromsø, Norway; University Paris Sud, France

## Abstract

**Background:**

Lupus nephritis is characterized by deposition of chromatin fragment-IgG complexes in the mesangial matrix and glomerular basement membranes (GBM). The latter defines end-stage disease.

**Methodology/Principals:**

In the present study we determined the impact of antibodies to dsDNA, renal Dnase1 and matrix metalloprotease (MMP) mRNA levels and enzyme activities on early and late events in murine lupus nephritis. The major focus was to analyse if these factors were interrelated, and if changes in their expression explain basic processes accounting for lupus nephritis.

**Findings:**

Early phases of nephritis were associated with chromatin-IgG complex deposition in the mesangial matrix. A striking observation was that this event correlated with appearance of anti-dsDNA antibodies and mild or clinically silent nephritis. These events preceded down-regulation of renal Dnase1. Later, renal Dnase1 mRNA level and enzyme activity were reduced, while MMP2 mRNA level and enzyme activity increased. Reduced levels of renal Dnase1 were associated in time with deficient fragmentation of chromatin from dead cells. Large fragments were retained and accumulated in GBM. Also, since chromatin fragments are prone to stimulate Toll-like receptors in e.g. dendritic cells, this may in fact explain increased expression of MMPs.

**Significance:**

These scenarios may explain the basis for deposition of chromatin-IgG complexes in glomeruli in early and late stages of nephritis, loss of glomerular integrity and finally renal failure.

## Introduction

A wide spectrum of autoimmune responses and organ manifestations are characteristic of Systemic lupus erythematosus (SLE), and are used by the American College of Rheumatology (ACR) as criteria to classify SLE. [1] Of particular importance in context of the present study are criteria linked to development of kidney disease: Production of potentially pathogenic anti-dsDNA antibodies (criterion # 10) and deposition of chromatin-containing immune complexes in kidneys (criterion # 7).

Over time, different concepts have been discussed to describe possible basic processes linked to initiation of lupus nephritis, and to progression of mild into end-stage organ disease. There is a consensus stating that anti-dsDNA and anti-chromatin antibodies are central in initiation and maintenance of lupus nephritis, but there is no agreement as to how they interact with glomerular structures. This could be due to cross-reaction of anti-chromatin antibodies with inherent glomerular structures like laminin [2]–[4], α-actinin [5]–[7], or with membrane components of mesangial cells [8], [9], or to binding of anti-chromatin antibodies to chromatin fragments exposed in affected glomeruli. Recent data favour the latter model.

We have demonstrated that chromatin fragments possess a high intrinsic affinity for glomerular membrane and matrix components like laminins and collagen IV [10]. These fragments are observed as electron dense structures (EDS) along glomerular basement membranes (GBM) and in the mesangial matrix. Glomerular EDS are terminal deoxynucleotidyl transferase-mediated dUTP nick end-labelling (TUNEL) positive, demonstrating that they contain nicked DNA [10], [11]. Furthermore, antibodies to components of chromatin, like those reactive with DNA, histones or transcription factors, bind in vitro to antigens present in EDS in murine [12], [10] and human [11] forms of lupus nephritis. Binding of antibodies in vivo to other structures that are not parts of EDS have not been observed in these studies [13].

It is not clear why chromatin fragments are exposed in kidneys, but this phenomenon may be linked to reduced ability of renal nucleases to degrade apoptotic or necrotic chromatin within the kidneys. We have recently demonstrated that reduced fragmentation of chromatin during evolution of nephritis concur with an acquired loss of renal *Dnase1* mRNA at the time when nephritis transforms into end-stage organ disease [14], [15]. Dnase1 accounts for more than 80% of total renal nuclease activity [16]. With low renal Dnase1 enzyme activity, apoptotic chromatin may not be appropriately fragmented and may instead be transformed into secondary necrotic chromatin fragments released from apoptotic blebs. Secondary to this event, chromatin fragments are exposed to the environment within kidneys, and bind glomerular membranes. Chromatin fragments may also be taken up by macrophages and dendritic cells in which they stimulate TLRs through CpG (TLR9) or RNA (TLR7) structures [17]–[23]. Engagement of TLRs may serve two functions: Up-regulation of co-stimulatory molecules (CD80/CD86) which are important for activation of chromatin-specific T helper cells [24], [25], required to transform chromatin-specific B cells into anti-chromatin antibody-secreting plasma cells; and up-regulation of certain matrix metalloproteases (MMPs) [22], [23]. Secreted MMPs have the potential to disintegrate GBM and the mesangial matrix by enzymatic degradation [26], [27]. This may facilitate deposition of chromatin fragment-IgG complexes in GBM. In harmony with this assumption, MMP2 and MMP9 activities are reported to be increased within glomeruli of nephritic, but not pre-nephritic, (NZBxNZW)F1 (BW) mice [28].

From data in these referenced studies, we predict that loss of renal Dnase1 correlates with exposure of large chromatin fragments within glomeruli and with increased MMP activities in the kidneys. The present study was designed to analyse how regulation of Dnase1, MMP2 and MMP9 mRNA levels and enzyme activities correlate with each other, with production of antibodies to dsDNA, with successive deposition of EDS in the mesangial matrix and in GBM, and finally with progressive proteinuria characteristic of lupus nephritis. The data presented here may explain the molecular and genetical basis for deposition of chromatin-IgG complexes in glomeruli in early and late stages of nephritis, with loss of glomerular integrity and renal failure as the final outcome.

## Results

### Dnase1, MMP2 and MMP9 mRNA Levels and Corresponding Enzyme Activities, and Degree of Proteinuria in Pre-Nephritic BW Mice and in Mice with EDS in the Mesangial Matrix or in GBM

**Figure 1:**
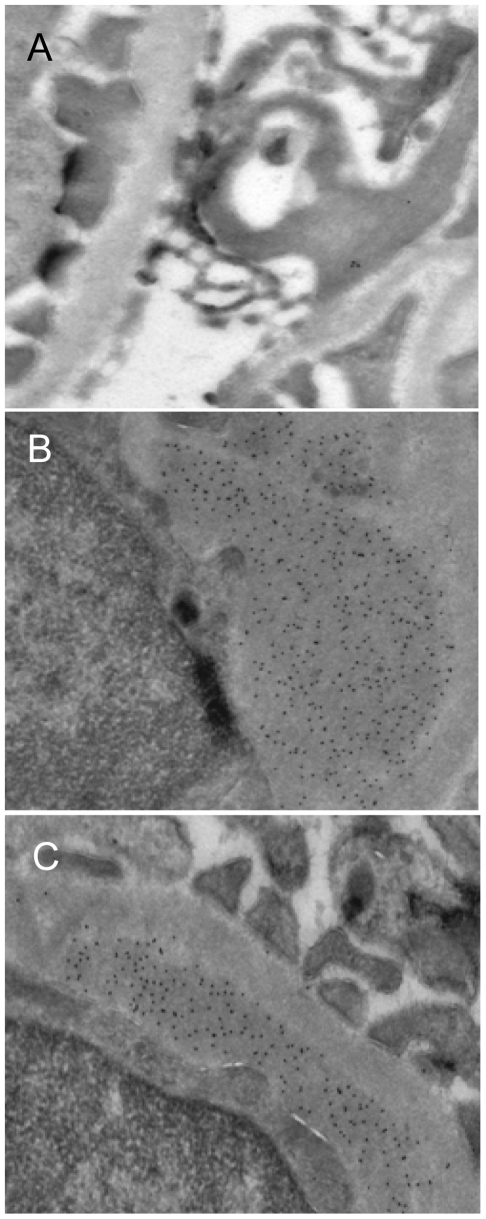
(NZBxNZW)F1 mice grouped according to glomerular location of EDS deposits. (NZBxNZW)F1 mice were sacrificed approximately every second week (in sets of 3) until development of end-stage lupus-like nephritis. The mice were sorted into 3 main groups according to kidney morphology; pre-nephritic mice (Group 1, no glomerular deposits of EDS (n = 27), A), mice with mesangial EDS deposits (Group 2 (n = 17), B), or mice with EDS deposits in GBM (Group 3 (n = 10), C). Magnification×40 k.

**Figure 2:**
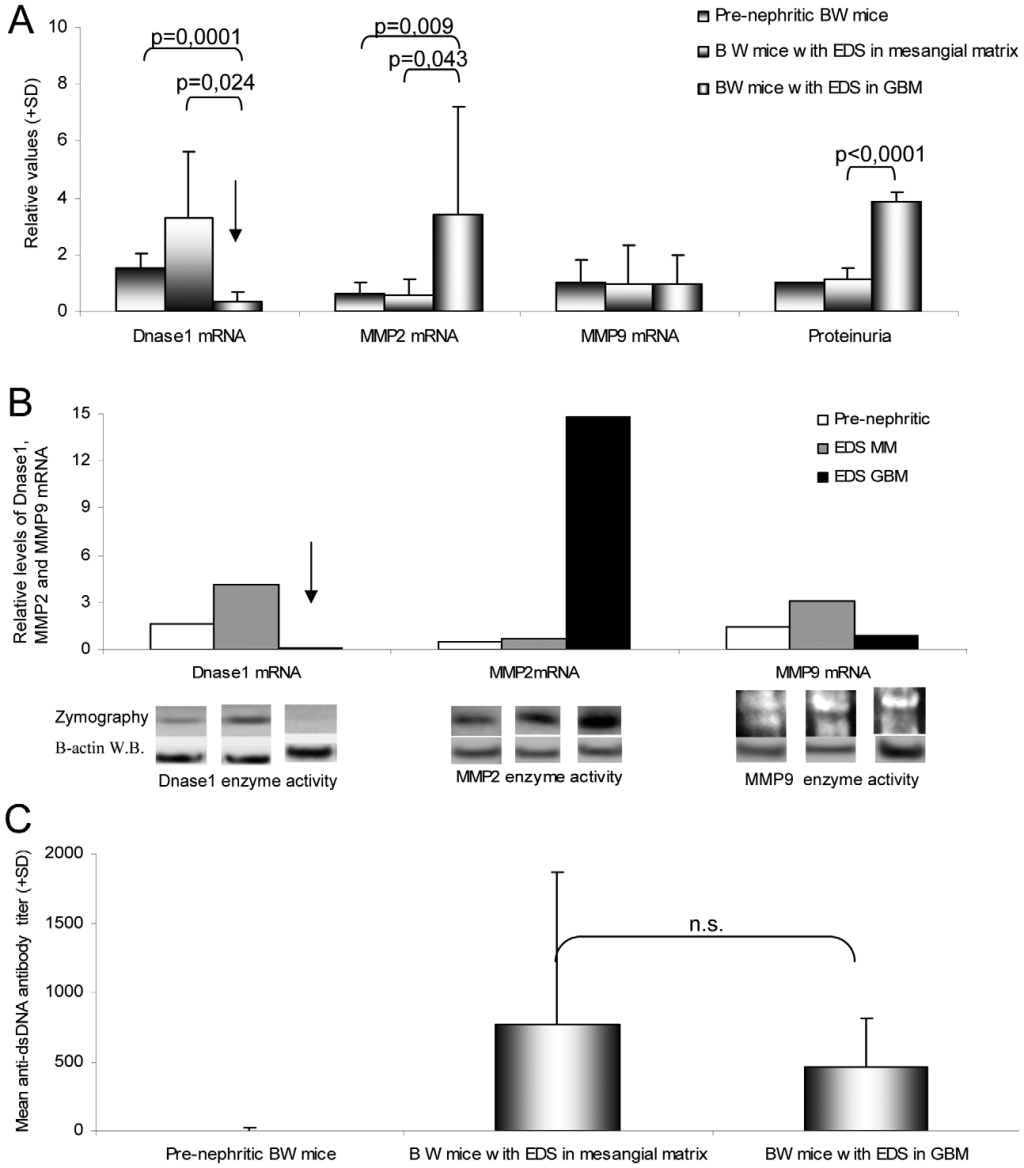
Levels of Dnase1, MMP2 and MMP9 mRNA and enzyme activities, and mean anti-dsDNA antibody titers in Group 1–Group 3 mice. There were no significant differences with respect to Dnase1, MMP2 or MMP9 mRNA levels and degree of proteinuria between Group 1 and Group 2 mice (A). In Group 3 mice, Dnase1 mRNA levels were severely and significantly down-regulated, while MMP2 mRNA levels and proteinuria were significantly higher than in the former 2 groups of BW mice (A). The variations in mRNA levels were reflected in a similar variation in enzyme activities, as demonstrated in individual mice by relevant zymography analyses (exemplified in B, see also [29]). The results in A and B indicate that low Dnase1, high MMP2 and severe proteinuria may be interdependent parameters. Mean anti-dsDNA antibody titers were lower in Group 3 mice, compared with mice in Group 2, although the difference was not statistically significant (C). Arrows in A and B point at reduced Dnase1 mRNA levels. Results are given as mean (±SD) and an unpaired t test was performed to determine differences between each group for each parameter. A one-way ANOVA was performed to compare all groups for each parameter. n.s.: not significant.

BW mice were sacrificed approximately every second week (in sets of 3) until development of end-stage lupus-like nephritis, and were analyzed for renal Dnase1, MMP2 and MMP9 mRNA levels and for corresponding enzyme activities. The mice were initially divided into 3 groups according to kidney morphology; pre-nephritic mice (Group 1, no glomerular deposits of EDS (n = 27), Figure 1A), mice with mesangial EDS deposits only (Group 2 (n = 17), Figure 1B), or mice with EDS deposits in both mesangial matrix and in GBM (Group 3 (n = 10), Figure 1C). The number of mice in these groups refers to mice with complete sets of data (n = 54, see Table S1 for complete sets of data in individual mice identifiable by labels). In analyses of individual parameters, 2 additional mice with relevant data are included. These were not analyzed for glomerular EDS deposits and are excluded from Figures 2, 4B, 4C, 5A, 5B and 6. No glomerular deposits of EDS were observed in sex and age matched BALB/c mice (data not shown, see also [12]).

As demonstrated in Figure 2A, there were no significant differences between Dnase1, MMP2 or MMP9 mRNA levels in mice from Group 1 and Group 2. In Group 3 mice, however, Dnase1 mRNA levels were severely down-regulated compared with mice in Group 1 (p = 0.0001) and Group 2 (p = 0.024). MMP2 mRNA levels were higher in Group 3 compared with Group 1 (p = 0.009) and Group 2 (p = 0.043) mice, while there were no statistically significant differences in MMP9 mRNA levels between the groups (Figure 2A). In Group 3 mice, proteinuria was significantly more pronounced than in the former 2 groups of BW mice (p<0,001, Figure 2A). The variations in Dnase1, MMP2, and MMP9 mRNA levels were reflected in a similar variation in enzyme activities, as demonstrated by relevant zymography analyses (exemplified in Figure 2B, see also [29]). For more extended analyses of mRNA levels and their correlation with enzyme activities, see Table S1 (for mRNA levels) and Figure S1 (for zymographic Dnase1 enzyme activities in individual mice). These results indicate that low Dnase1 activity, high MMP2 activity, and severe proteinuria are interdependent parameters involved in development of end-stage kidney disease. Since mRNA levels are given as exact figures, and correlate with zymography results (Table S1 and Figure S1), which, however, are not given as exact measures, data in the next sections are given as fold change in Dnase1 and MMP mRNA levels.

Although not statistically significant, mean anti-dsDNA antibody titers were lower in Group 3, compared with Group 2 mice (Figure 2C). In sex and age matched BALB/c mice, Dnase1, MMP2 and MMP9 mRNA levels and enzyme activities were present at normal and stable values throughout the observation time (See Figure S1 for Dnase1 zymography using proteins extracted from BALB/c kidneys). This has also been demonstrated in a series of descriptive observations [14], [15], [29]. All these mice were negative for anti-dsDNA antibodies (data not shown).

### Loss of Renal Dnase1 mRNA Correlates with Increased Renal MMP2 mRNA Levels

**Figure 3:**
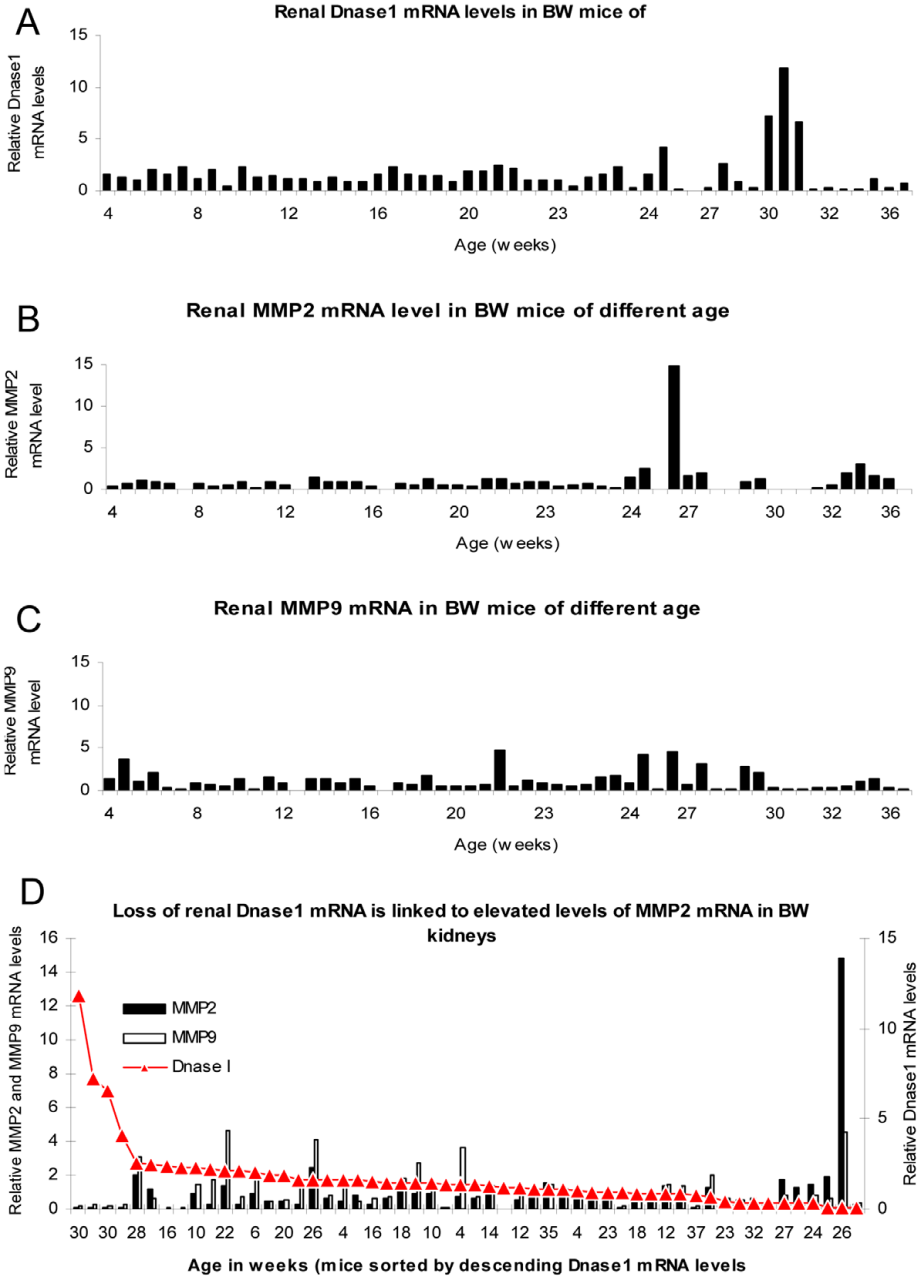
Dnase1, MMP2 and MMP9 mRNA levels and their correlations in mice of different age. Dnase1 mRNA levels fluctuated over the entire observation time (A). Similarly, both MMP2 and MMP9 showed a considerable variability with some peaks in mice of higher age (B and C, for MMP2 and MMP9, respectively). To analyse if levels of Dnase1, MMP2 and MMP9 mRNA correlated with each other, the data were combined for each mouse, and this set of data was sorted by descending Dnase1 mRNA levels. As demonstrated in panel D, very low levels of Dnase1 were inversely correlated with high levels of MMP2 (correlation coefficient −0,299, p = 0,016). This indicates that there is a link between loss of Dnase1 and up-regulation of MMP2. Chromatin fragments that are not appropriately fragmented and cleared may be the common denominator (see text for details).

Dnase1 mRNA levels varied considerably in mice analyzed at various time points over the entire observation period (Figure 3A). Similarly, both MMP2 and MMP9 mRNA levels showed a substantial variability with some peaks generally in mice of older age (Figure 3B and 3C, for MMP2 and MMP9 mRNA, respectively). Since nephritis does not appear at the same age in individual BW mice, instead of relating data to age, levels of Dnase1, MMP2 and MMP9 mRNA were combined for each mouse, and this set of data was sorted by descending Dnase1 mRNA levels. As is clearly demonstrated in Figure 3D, very low levels of Dnase1 correlated inversely with high levels of MMP2 (Table 1, correlation coefficient = −0,299, p = 0.016). This was also demonstrated by a similar inverse correlation of Dnase1 and MMP2 enzyme activities (Figure 2B and Table S1 and Figure S1, which demonstrates that mRNA levels largely reflected enzyme activities). This indicates that there may be a causal relationship between loss of Dnase1 and up-regulation of MMP2 (see below). There was no significant correlation between Dnase1 and MMP9 mRNA levels (Figure 3D, Table 1).

**Table 1:** Statistical analyses of the parameters analyzed: Correlations and significances.^1^

	Age	Dnase1	MMP2	MMP9	EDS^2^ MM^3^	EDS GBM^4^	Anti-DNA abs^5^	Proteinuria
Age	-	−0.220	0.052	−0.166	0.783	0.519	0.754	0.643
Dnase1	**0.057**	-	−0.299	0.018	0.051	−0.565	−0.124	−0.693
MMP2	**0.500**	**0.016**	-	0.719	−0.029	0.345	0.138	0.227
MMP9	**0.271**	**0.911**	**<0.001**	-	−0.130	0.028	−0.149	−0.078
EDS MM^6^	**<0.001**	**0.740**	**0.850**	**0.399**	-	0.477	0.899	0.450
EDS GBM	**<0.001**	**<0.001**	**0.010**	**0.835**	**<0.001**	-	0.456	0.836
Anti-DNA abs	**<0.001**	**0.124**	**0.221**	**0.333**	**<0.001**	**0.001**	-	0.540
Proteinuria	**<0.001**	**<0.001**	**0.062**	**0.589**	**0.002**	**<0.001**	**<0.001**	-

1 Upper diagonal part contains correlation coefficient estimates, lower diagonal part (in bold) contains corresponding p-values.

2 EDS: electron dence structures.

3 MM: mesangial matrix.

4 GBM: glomerular basement membranes.

5 abs: antibodies.

6 Correlation and corresponding p-values for EDS in mesangial matrix are calculated from mice with no glomerular EDS, or EDS in the mesangial matrix only. In these calculations, we excluded mice with EDS in GBM, since all these have mesangial matrix deposits, while the biological consequences of EDS in GBM overrule those of EDS in the mesangial matrix (see text).

### Proteinuria Is Not Related to Serum Anti-dsDNA Antibody Titers, but Correlates with EDS in the GBM, and Inversely with Renal Dnase1 mRNA Levels

**Figure 4:**
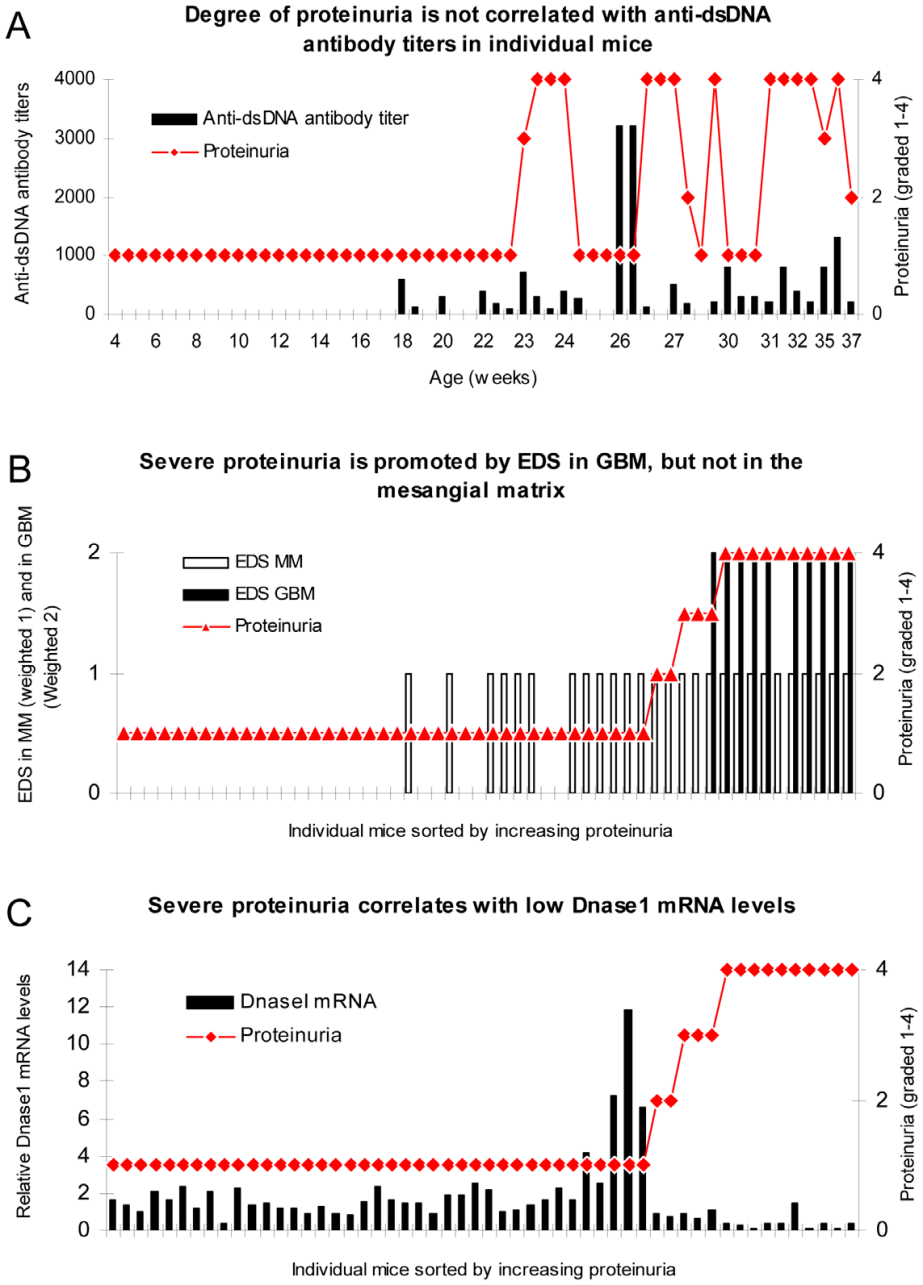
Severe proteinuria correlates with EDS deposits in GBM, and inversely with renal Dnase1 mRNA levels. In mice sorted for age, there was no association between degree of proteinuria and levels of anti-dsDNA antibody titers (A). To analyse if location of EDS deposits had impact on proteinuria, data on proteinuria and deposition of EDS in the mesangial matrix (weighted 1 in B) or in the GBM (weighted 2 to make a visual distinction from deposits in the mesangial matrix) were combined for each mouse, and sorted by ascending values of proteinuria. Severe proteinuria (≥20 g/L) was, except for one mouse with intermediate proteinuria (≤3 g/L), exclusively observed in mice with EDS in GBM (B), while intermediate or mild proteinuria was observed in only 4 out of 17 mice with mesangial matrix deposits (B). In panel C, degree of proteinuria and renal Dnase1 mRNA levels were paired and sorted by ascending proteinuria. Severe proteinuria (≥20 g/L) correlated with a substantial loss of Dnase1 mRNA (and enzyme activity, see Figure 2). Thus, in mice selected for proteinuria ≥20 g/L, renal Dnase1 mRNA was nearby lost in all but one mouse (C), and deposits of chromatin-IgG complexes (observed as EDS) in GBM were observed only in these mice. For statistics, see Table 1.

In order to examine the impact of anti-dsDNA antibodies on development of proteinuria, we analyzed if the presence of anti-dsDNA antibodies correlated with proteinuria. These parameters correlated with each other (p<0,001, Table 1), while titers of the antibodies were not associated with degree of proteinuria as high antibody titers appeared in individual mice with low degree of proteinuria and vice versa (Figure 4A).

Next, data on proteinuria and deposition of EDS in either the mesangial matrix only (weighted 1 in Figure 4B) or also in the GBM (weighted 2 to make a visual distinction from deposits in the mesangial matrix in the figure) were combined for each mouse, and the paired data were sorted by ascending values of proteinuria. Figure 4B shows that severe proteinuria (≥20 g/L) was, except for one mouse, exclusively observed in mice with EDS in GBM, while intermediate or low levels of proteinuria was observed in 4 out of the 17 mice with mesangial matrix deposits only, which is a significantly higher number than for mice without any EDS deposition in glomeruli (0 out of 27 mice, see Table 1 for statistics). The data also demonstrate that deposition of chromatin fragment-IgG complexes in the mesangial matrix may not necessarily result in clinical nephritis. EDS deposits in the mesangial matrix may, therefore, be compatible with a silent organ manifestation in individual mice.

To further investigate the hypothesis that chromatin fragment deposition, especially in the GBM, depends on loss of renal Dnase1, we assessed whether severe proteinuria indeed correlated inversely with Dnase1 mRNA levels and Dnase1 activity the same way as severe proteinuria correlated with EDS in GBM. When proteinuria and Dnase1 mRNA levels were combined in individual mice, and the two parameters sorted by ascending proteinuria, it became evident that severe proteinuria correlated with critically down-regulated Dnase1 (Figure 4C, p<0.001, Table 1). Thus, in mice selected on the basis of proteinuria ≥20 g/L, renal Dnase1 mRNA was nearly absent in all but one mouse (Figure 4C), and deposits of chromatin-IgG complexes (observed as EDS) in GBM were observed only in these mice except for one with proteinuria of 3 g/L (Figure 4B).

### Presence of Anti-dsDNA Antibodies and Low Renal Dnase1 mRNA Level and Corresponding Enzyme Activity Correlate with the Deposition of EDS in the Mesangial Matrix and in the GBM, Respectively

**Figure 5:**
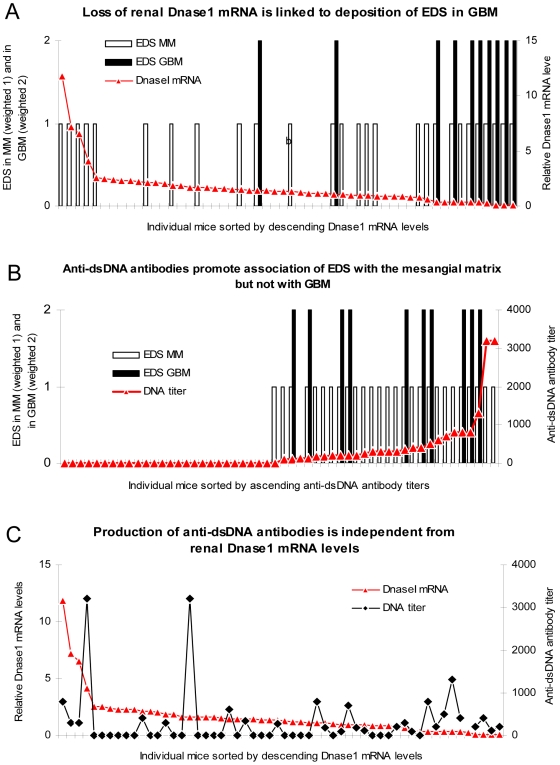
Anti-dsDNA antibodies and renal Dnase1 levels, and their correlation with EDS in mesangial matrix and GBM, respectively. Data on Dnase1 mRNA levels and EDS in the mesangial matrix (weighted 1 in Figure 5A and B), or in GBM (weighted 2) where combined for each mouse and sorted by descending Dnase1 mRNA levels. The result of this analysis demonstrates a clear negative correlation between Dnase1 mRNA levels and presence of EDS in GBM. This association was statistically highly significant (A, see Table 1 for statistical analyses). The inverse correlation of EDS in the mesangial matrix with Dnase1 mRNA levels was weaker, and did not reach statistical significance (A, Table 1). The presence of EDS solely in the mesangial matrix was significantly associated with production of anti-dsDNA antibodies (B, Table 1). This demonstrates that deposition of EDS in the mesangial matrix and in the GBM may originate from different molecular processes; EDS in mesangial matrix depend on presence of antibodies to dsDNA, while EDS in GBM depend on reduced renal Dnase1 activity. Production and titers of antibodies to dsDNA was not significantly associated with levels of renal. Dnase1 mRNA and enzyme activities (C, Table 1).

According to the data presented in Figure 4, which demonstrate that severe proteinuria is inversely correlated with Dnase1 mRNA levels and enzyme activities, and positively with EDS in GBM, it was important to analyse whether low Dnase1 mRNA levels also correlated with EDS deposition in the mesangial matrix, or only in GBM. Data on Dnase1 mRNA levels and EDS in mesangial matrix (weighted 1 in Figure 5A and B), or in GBM (weighted 2) where combined and sorted by descending Dnase1 mRNA levels. The result of this analysis is presented in Figure 5A, and demonstrates a clear correlation between low Dnase1 mRNA levels and the presence of EDS in GBM. Loss of Dnase1 mRNA is also reflected by a corresponding loss of enzyme activity (see Figure 2B and Figure S1 for representative examples). The association of low Dnase1 mRNA levels and presence of EDS in GBM was statistically highly significant (correlation coefficient = −0,565, p<0,001, Table 1). EDS in the mesangial matrix was not associated with Dnase1 mRNA levels (Figure 5A, p = 0,740, Table 1)). The latter deposits are, therefore, most likely generated by a mechanism independent from renal Dnase1 activity.

The presence of EDS solely in the mesangial matrix was significantly associated with the presence of anti-dsDNA antibodies (Figure 5B, correlation coefficient = 0,899, p<0,001, Table 1). This demonstrates that deposits of EDS in the mesangial matrix and in the GBM have different origins; EDS in the mesangial matrix may depend on the presence of antibodies to dsDNA, while deposition of EDS in GBM may be a consequence of reduced renal Dnase1 activity. On the other hand, production of antibodies to dsDNA did not correlate with the levels of renal Dnase1 mRNA and enzyme activities (Figure 5C, p = 0,124, Table 1).

### Overall Statistical Analyses

**Figure 6:**
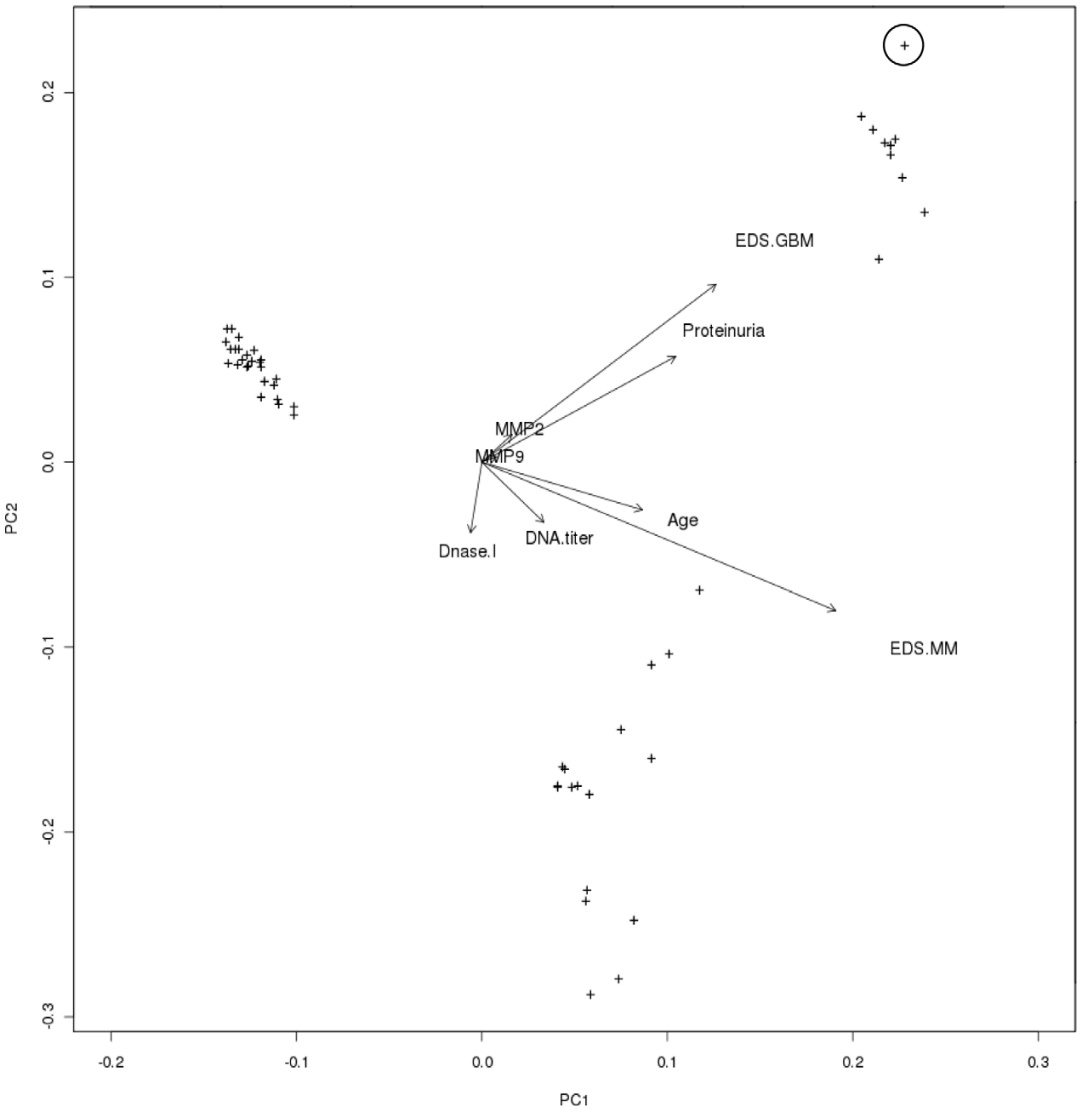
A principal component analysis (PCA) of parameters included in this study. This PCA biplot aims to optimally display variances and not correlations. The angles between the various biplot axes serve as good indicators of the correlations among the variables (shown as arrows). Similarly, the position of the samples of individual mice (shown as plus signs) relative to the arrows, provide good indications as to which variable(s) have had the largest effect. The result of the biplot demonstrates that groups emerging from this analysis perfectly correlated with the groups of BW mice as defined in Figure 1 and Figure 2, defined as pre-nephritic BW mice (Group 1), BW mice with deposits of EDS in the mesangial matrix (Group 2) or with deposits in the GBM (Group 3). The circle identifies the mouse with the lowest renal Dnase1 mRNA level and enzyme activity, and the highest MMP2 and MMP9 mRNA levels and enzyme activities and with proteinuria ≥20 g/L.

Table 1 shows a square matrix where the upper diagonal part demonstrates correlation coefficients, and the lower diagonal part the corresponding p-values of the data generated in this study. In Figure 6, the result of a principal component analysis (PCA) biplot drawn with the R biplot function is demonstrated. The PCA biplot is aimed to optimally display variances and not correlations. The angles between the various biplot axes are good indicators of the correlations among the variables (shown as arrows). The position of the samples of individual mice (shown as plus signs) relative to the arrows provides good indications as to which variable(s) have had the largest effect. As is evident in data presented in Figure 6, the mice confine perfectly into three groups, one pre-nephritic, one with mild mesangial nephritis, and one with end-stage membrano-proliferative nephritis (see Figure S2 to identify the grouped mice in Figure 6 by labels). This result confirms that the parameters used to group BW mice as in Figure 1 and Figure 2 are biologically relevant.

## Discussion

The basic hypothesis motivating the present study was that clinical lupus nephritis is a distinct organ disease with an aetiology linked to an acquired loss of renal Dnase1 enzyme activity. Acquired deficiency of renal Dnase1 activity is assumed to promote a progressive exposure of secondary necrotic chromatin in GBM, and a consequent development of severe nephritis [30], [31]. This may, however, be restricted to individuals with anti-chromatin antibodies. In the absence of anti-chromatin antibodies, exposed chromatin may be more or less harmless. Similarly, antibodies in the absence of exposed chromatin may render them apathogenic. This latter statement relates to their pathogenic [30], [31], but not to their diagnostic impact [32]. The lupus nephritis phenotype is, therefore, characterized by glomerular binding of complexes of chromatin fragments and anti-chromatin antibodies.

Data so far demonstrate that accumulation of chromatin fragment-IgG complexes correlates with a progressive loss of renal Dnase1 enzyme activity [14] and with increased renal MMP2 and, to a lesser extent, MMP9 activities [28], [29]. MMPs may disrupt and disintegrate mesangial matrix and GBM [26], [27]. These two events explain why large chromatin fragments generated within the kidneys finally get access to GBM. Therefore, loss of Dnase1 and increased MMP activities are identified as factors that may contribute to transformation of mild mesangial into severe membrano-proliferative lupus nephritis.

In this study we analyzed if loss of renal Dnase1 correlated with increased MMP activity in the kidneys, and with exposure of large chromatin fragments at loci typical for lupus nephritis–namely in the mesangial matrix and in the GBM. A series of baseline data were collected in groups (n = 3) of mice at consecutive intervals. These were combined to analyse if regulation of Dnase1, MMP2 and MMP9 mRNA levels and enzyme activities (analyzed by relevant zymography assays) correlated with the production of antibodies to dsDNA, and with the successive deposition of EDS in the mesangial matrix and in GBM in individual mice. In the end, these factors were correlated with progressive proteinuria and end-stage organ disease.

For each parameter, it was difficult to determine correlation between e.g. Dnase1, MMP2 or MMP9 mRNA levels with age in individual mice since nephritis developed at different time points in different mice. However, by combining data obtained in each mouse, and by sorting them by parameters one by one at the time in an ascending or descending way, clear correlations became apparent. For example, a combination of the highest values for MMP2 combined with chromatin fragments accumulated in GBM were only observed in mice with the lowest renal Dnase1 mRNA levels. This indicates that these kidneys have entered the state of end-organ disease when Dnase1 mRNA and enzyme activity were at the lowest levels. Similarly, sorting data by increasing proteinuria, it became evident that mice with severe proteinuria had the lowest levels of renal Dnase1. These observations fit with the hypothesis that reduced Dnase1 mRNA level and enzyme activity result in reduced fragmentation of chromatin from dead cells. Large chromatin fragments became, instead of being cleared, retained in tissue, and exposed to infiltrating dendritic cells and macrophages. Upon interaction of chromatin with TLRs in these cells, co-stimulatory molecules (CD80/CD86) are up-regulated, while peptides from the same chromatin fragments may get processed, and presented by MHC class II molecules. This may, although not proven by data, be sufficient to activate chromatin-specific T cells with potential to transform chromatin-specific B cells into antibody-secreting plasma cells [21], [30], [33].

On the other hand, chromatin fragments that are not appropriately fragmented and cleared may be the factor that determines if induced anti-chromatin antibodies gain pathogenic potential. This can only happen if chromatin is exposed and thereby made available for such antibodies. One factor that can exaggerate this situation is increased secretion of MMPs, since chromatin fragments have the potential to up-regulate MMP production and secretion through activation of the TLR9 signaling pathway [22], [23], [34], [35]. This does not rule out that other mechanisms may contribute to increased production of MMPs in lupus nephritis (see [36], [37] for reviews). Persistently increased MMP activity within glomeruli may, therefore, be a result of an inflammatory process maintained by retained necrotic cellular debris linked to loss of apoptosis- and necrosis-related Dnase1 enzyme activity [38], [39]. Continuously increased matrix degradation by the MMPs may disrupt GBM integrity and thereby promote deposition of immune complexes in these structures, as discussed by Tveita et al. [29]. This process is reflected by the data presented in this study, where loss of Dnase1 correlated with increased MMP2 mRNA levels and enzyme activities in affected kidneys; with increased exposure of chromatin fragments in complex with IgG within GBM; and with severe proteinuria (≥20 g/L). These results create the basis for similar studies in human lupus nephritis. Preliminary results from analyses of kidney biopsies from patients with human lupus nephritis demonstrate a similar relationship between severe nephritis and loss of the Dnase1 enzyme (studies in progress).

Since deposition of EDS in the mesangial matrix preceded reduced renal Dnase1 levels, this less harmful process has another origin. In the data sets presented here, it is evident that deposition of EDS in the mesangial matrix correlated significantly with appearance of anti-dsDNA antibodies in sera. An explanation for this linkage may be that circulating nucleosomes [40]–[42] bind nucleosome-reactive antibodies, and re-circulate as immune complexes. These may be bound to glomerular mesangial cells through Fc regions of IgG in the complexes, since these cells express Fcγ-receptors [43]. If the amount of immune complexes exceeds the clearance capacity of mesangial cells, this could result in release of immune complexes into the mesangial matrix surrounding these cells.

The consequent interpretation of the data presented here is that lupus nephritis is a principally two-stepped organ disease where each step has its distinct aetiology. The early phase of lupus nephritis correlates with deposition of complexes of chromatin fragments and IgG in the mesangial matrix. This process is associated with the production of anti-DNA (anti-chromatin) antibodies, and is characterized by mild or clinically silent nephritis. This process is consistent with an observed drop in serum concentration of DNA (nucleosomes) at the time when anti-dsDNA antibodies appeared in circulation, as determined by real time PCR applied to serial serum samples from BW mice or to sera from human SLE patients. This real time PCR was performed with Alu- (human) or B1- (mouse) specific primers (M Hellvik Jørgensen et al., manuscript in preparation).

At a certain time point in the life of BW mice, the renal Dnase1 mRNA and enzyme activity is inevitably lost. Importantly, reduced renal Dnase1 activity manifests itself at the same time fragmentation of chromatin in kidneys is reduced. The reason for the loss of renal Dnase1 is uncertain, and may involve different processes. Alteration of the Dnase1 promoter (promoter methylation, [44]), and the effect of regulatory RNA (like microRNA, [45]) are likely and testable processes. Of particular interest, however, is the fact that the hsp90-related protein Trap1 is encoded the opposite direction of Dnase1, and uses down-stream sequence elements of the Dnase1 gene (see http://genome.ucsc.edu). This means that the two genes may not be transcribed at the same time, and therefore, they may mutually exclude each other. Trap1 is up-regulated during stress and has the function of a survival protein [46], [47]. Thus, Dnase1, as a death-associated protein, and Trap1 have antagonistic effects, and possibly also antagonistic expression profiles. These aspects are currently under investigation in our laboratory.

To understand how the Dnase1 gene is down-regulated in the kidney may bring us a significant step towards the understanding of the molecular and genetical events that in the end result in progressive lupus nephritis. This insight is decisive to create a basis for development of new causal therapy modalities.

## Materials and Methods

### Ethics Statement

The National Animal Research Authority (NARA) approved the study. Treatment and care of animals were conducted in accordance with guidelines of the Norwegian Ethical and Welfare Board for Animal Research.

### Murine Tissue Samples

Renal tissue was collected from female (NZBxNZW)F1 (BW) and female age-matched BALB/c mice (Jackson Laboratory, Bar Harbor, Maine, USA) sacrificed approximately every second week (n = 3) from the age of 4 weeks until development of end-stage disease in the BW mice, clinically defined when severe proteinuria developed (≥20 g/L). Tissue was either snapfrozen for protein extraction, preserved according to Tokuyasu for immune electron microscopy [48], or preserved in RNAlater (Ambion Inc., Texas, USA) for mRNA analyses. Serum and urine samples were collected at 2–3 week intervalls and stored at −80°C.

### Determination of Proteinuria

Proteinuria was determined by urine stix (Bayer Diagnostics, Bridgend, United Kingdom): 0–1+ (≤0.3 g/L, regarded as physiological proteinuria); 2+, (≤1 g/L); 3+, (≤3 g/L); and 4+, (≥20 g/L).

### Anti-dsDNA Antibody ELISA

Serum anti-dsDNA antibodies were detected and controlled by ELISA as described [32], [49], using microtiter plates (Nunc MaxiSorp; Nunc, Copenhagen, Denmark) coated with calf thymus dsDNA (10 µg/ml in PBS, Sigma-Aldrich, Saint Louis, USA).

### Immune Electron Microscopy (IEM)

For immune electron microscopy, kidney samples were fixed in 8% formaldehyde in PBS, further processed in sucrose and glycine before mounted and immersed in liquid nitrogen as described [48]. Ultrathin cryosections were processed as described [12] to correlate the morphological changes with the presence of autoantibodies bound in the glomeruli in vivo. The grids were contrasted with uranyl acetate and examined at ×20–×40 K magnification using a JEM-1010 transmission electron microscope (Jeol, Tokyo, Japan).

### Purification of Renal RNA and cDNA Synthesis

Total RNA was isolated from RNAlater-preserved kidneys using EZ-1 RNA tissue mini kit (Qiagen, Nordic, Norway). The kidneys were taken from all mice included in this study. These are listed in Table S1. The concentration of extracted RNA was determined by spectrophotometry using NanoDrop (NanoDrop technologies, Wilmington, USA), and quality was assessed using Agilent Bioanalyzer (Agilent Technologies, Santa Clara, USA). Samples were reverse-transcribed with random primers using High Capacity cDNA Reverse Transcription kit (Applied Biosystems, Foster City, USA).

### Real-Time PCR Analysis

For real time PCR we used TaqMan® Gene Expression Assays (Applied Biosystems, CA, USA): Dnase1 Mm01342389_g1; MMP2 Mm00439508_m1; MMP9 Mm00442991_m1; endogenous control–Mouse ACTB (actin, beta) 4352933E and TATA binding protein Mm00446973_m1. The assays were performed on ABI Prism 7900HT Sequence Detection System (Applied Biosystems). Expression levels were calculated using the ΔΔCT method. Data are given as fold change compared to transcription in 4 weeks old mice.

### Purification of Renal Proteins

Proteins were purified from kidneys according to Woessner [50] as described by Tveita et al. [28]. Briefly, snapfrozen cortical tissue was homogenized in Tris buffer (0.5 mol/l Tris-HCl, pH 7.5, 150 mmol/l NaCl). The mixture was centrifuged and the supernatants were collected. The pellets were resuspended in heat extraction buffer (50 mM Tris pH 7.5, 0.1 M CaCl2, 0.15 M NaCl), incubated for 4 min at 60°C, and centrifuged. The supernatants were combined, and protein content was determined using BCA assay kit (Pierce Biochemicals, IL, USA). These preparations were used for Dnase1, MMP2 and MMP9 gel zymography.

### Dnase1 and MMP Gel Zymography

DNA degrading activity by Dnase1 was determined after separation of renal proteins in a 10% SDS-polyacrylamide gel containing 100 µg/ml heat-denatured salmon sperm DNA (Invitrogen Corp., Carlsbad, CA) as described [51]. MMP2 and MMP9 zymography was performed as described by Tveita et al. [29], as follows. After protein purification, proteins were separated on 7.5% SDS-PAGE gels containing gelatin (1.0 mg/ml). Following electrophoresis, gels were washed for 1 h in 2% Triton X-100 in 50 mM Tris-HCl, pH 7.4, followed by incubation at 37°C for 20 h in activation buffer (50 mM Tris-HCl, pH 7.4, 5 mM CaCl_2_). Gels were stained with Coomassie Blue. Clear zones against the background demonstrated presence of protease activity, and MMP2 and MMP9 were detected by MW according to position of recombinant enzymes in the gel. An actin-specific Western blot was performed to ensure equal loading of the different renal protein preparations using a rabbit IgG anti-actin antibody (Sigma-Aldrich).

### Statistics

Data are presented as mean with standard deviation (SD). An unpaired t-test was performed to test differences between each group and a one-way ANOVA was performed to compare all groups for each parameter; p<0.05 was considered significant. The rcor.test function from the R language ltm package was used to generate data in Table 1. All observations were included and Spearman was used for significance testing. A principal component analysis (PCA) was performed on the same set of data and a biplot drawn with the R biplot function (Figure 6).

## Supporting Information

Table S1 Summary of data on mRNA levels of Dnase1, MMP2, and MMP9, anti-dsDNA antibody titers, deposits of immune complexes in the mesangial matrix and GBM, and proteinuria linked to each individual (NZBxNZW)F1 mice which are identified by labels.
(0.03 MB XLS)

Figure S1 Dnase1 gel zymography is given for each (NZBxNZW)F1 and BALB/c mouse included in the study. This figure, combined with the Dnase1 mRNA levels presented in Table S1 demonstrate that levels of Dnase1 mRNA correspond with levels of Dnase1 enzyme activity.
(1.99 MB TIF)

Figure S2 This figure is identical to Figure 6 in the manuscript, with the additional information that each mouse can be identified by the same labels as in Table S1. The result of the biplot demonstrates that groups emerging from this analysis perfectly correlated with the groups of BW mice as given in Figure 1 and Figure 2, defined as pre-nephritic BW mice (Group 1), BW mice with deposits of EDS in the mesangial matrix (Group 2) or with deposits in the GBM (Group 3).
(0.11 MB TIF)
