# Supplementary figures and images for: Anti-dsDNA Antibodies Promote Initiation, and Acquired Loss of Renal Dnase1 Promotes Progression of Lupus Nephritis in Autoimmune (NZBxNZW)F1 Mice
Source: PLoS One. 2009 Dec 29;4(12):e8474. doi: 10.1371/journal.pone.0008474 (PMC2793523; doi:10.1371/journal.pone.0008474)

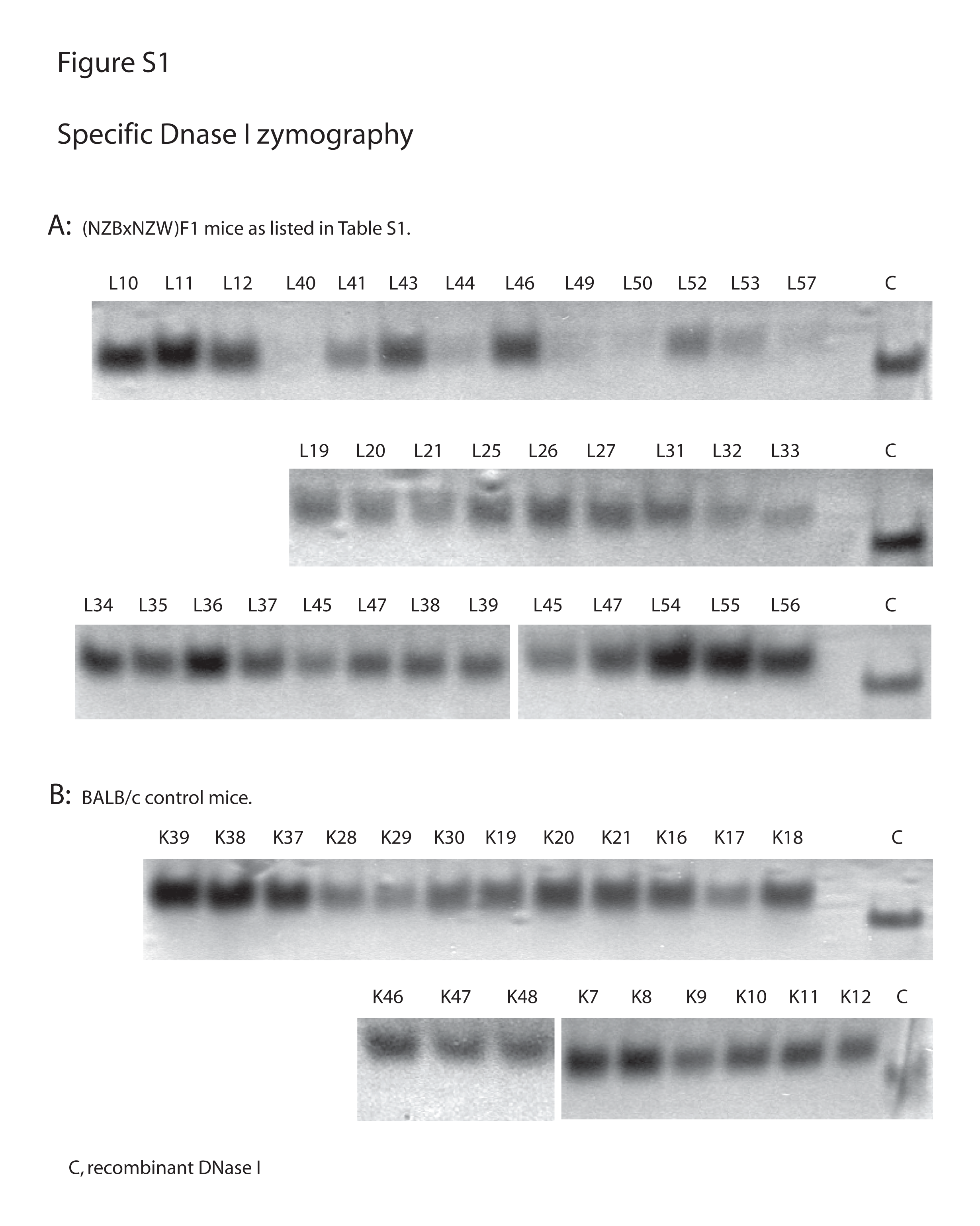

Supplement: Figure S1 — Dnase1 gel zymography is given for each (NZBxNZW)F1 and BALB/c mouse included in the study. This figure, combined with the Dnase1 mRNA levels presented in Table S1 demonstrate that levels of Dnase1 mRNA correspond with levels of Dnase1 enzyme activity. (1.99 MB TIF) [file pone.0008474.s002.tif]

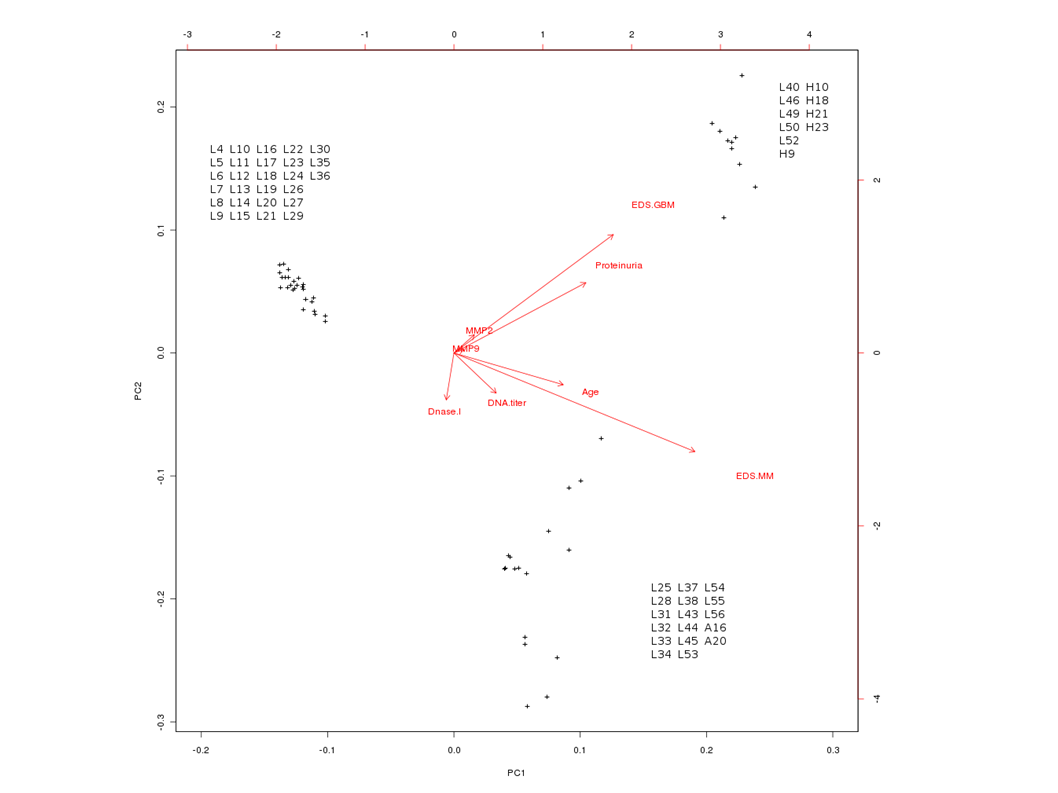

Supplement: Figure S2 — This figure is identical to Figure 6 in the manuscript, with the additional information that each mouse can be identified by the same labels as in Table S1. The result of the biplot demonstrates that groups emerging from this analysis perfectly correlated with the groups of BW mice as given in Figure 1 and Figure 2, defined as pre-nephritic BW mice (Group 1), BW mice with deposits of EDS in the mesangial matrix (Group 2) or with deposits in the GBM (Group 3). (0.11 MB TIF) [file pone.0008474.s003.tif]
